# Focusing on malignancies attributable to metabolic risk: analysis of global burden database and projections of future trends

**DOI:** 10.1097/JS9.0000000000003298

**Published:** 2025-08-25

**Authors:** Jiazhao Song, Jianke Ma, Saisai Jing

**Affiliations:** a Department of Oncology, The Affiliated Cixi Hospital, Wenzhou Medical University, Cixi, Zhejiang, China; b Experimental Tumorpathology, University Hospital Erlangen, Friedrich-Alexander-Universität Erlangen-Nürnberg, Erlangen, Germany

**Keywords:** cancer, Global Burden of Disease, malignancies, metabolic risk

## Abstract

**Background:**

Malignant tumors due to high metabolic risk are currently a major health threat worldwide, and their rising mortality rate poses a great challenge to cancer prevention and control. This study is based on data from the Global Burden of Disease 2021 to analyze trends in cancer deaths and predict future burdens.

**Methods:**

The burden of disease was assessed by aggregating age-standardized mortality rates and disability-adjusted life years (DALYs). Analyze the correlation between socio-demographic indices (SDIs) and gender and cancer incidence. Calculate the average annual percentage change (AAPC) and its corresponding 95% uncertainty interval to summarize the overall trend. Autoregressive integrated moving average (ARIMA) models were developed to predict future trends.

**Results:**

Cancer mortality from malignant tumors due to high metabolic risk has been trending upward each year over the past 30 years in middle SDI, low-middle SDI, and low SDI areas. The highest growth rates of increase were 51% in the low-middle SDI region, the AAPC values of 2.488 and 2.480 deaths and DALYs per 100 000 population, respectively. Cancer mortality rates in the medium-high SDI region decreased slightly after 2019. The ARIMA model predicts that mortality and DALYs will continue to rise steadily through 2050.

**Conclusion:**

The findings suggest the need for targeted health promotion outreach and clinical interventions supported by sustained medical inputs to more effectively manage the growing global cancer burden.

## Introduction

The increasing number of cancer-related deaths worldwide poses a significant threat to human health^[1]^. In the context of modern society’s rapid development, metabolic risk factors, such as high body mass index (BMI) and high fasting plasma glucose (FPG), are significantly high-risk triggers for cancer^[2,3]^. Patients with obesity and diabetes have a higher risk of cancer-specific mortality compared to non-obese and non-diabetic patients^[2,3]^. This suggests we should focus on assessing and monitoring these high-risk groups in our public policy guidelines for scientific cancer prevention and control. We should also respond to the increasing cancer mortality by developing comprehensive public health promotion and control measures. Based on large population-based data from the Global Health Database, we systematically analyzed the relevant characteristics of malignancies attributed to metabolic risk worldwide between 1990 and 2021, making trend projections of cancer development over the next 30 years, and addressing the shortcomings of previous studies^[4]^. Overall, our findings may provide a valuable reference for the precise prevention and control of malignant tumors related to high metabolic risk.

**HIGHLIGHTS**

- Globally, mortality and disability-adjusted life years (DALYs) from metabolically attributable cancers are on the rise.
- Central Europe has the highest mortality and DALYs. Other countries and regions with high socio-demographic indices, such as the high-income regions of North America and Western Europe, also generally have higher age-standardized rates for both mortality and DALYs.
- Global mortality and DALYs attributable to metabolically related cancers are projected to continue their increasing trend through 2050, with significant performance among middle-aged and older populations.

## Methods

The data for this study come from the Global Burden of Disease (GBD) 2021, which compiles nutritional health information from 1990 to 2021 for 204 countries and territories. These detailed data include cancer incidence, mortality, and disability-adjusted life years (DALYs). Age-standardized rates (ASRs) and cumulative change were calculated to elucidate temporal trends over the past three decades. In addition, future trends were projected by using an autoregressive integrated moving average (ARIMA) model. The association between cancer burden and socio-demographic index (SDI) was also examined. A joint point regression model was used to calculate annual average percentage changes (AAPCs) and their corresponding 95% uncertainty intervals (UIs), summarizing trend data from 1990 to 2021. Differences in population composition were avoided by quantifying metabolic risk mortality and age-standardized DALYs per 100 000 population. The Bayesian age-part cohort (BAPC) was used to project trends in malignancy incidence from 2021 to 2050. This study is original from the authors and was evaluated according to the TITAN Guidelines^[5]^.

## Results

### Trends in the burden of disease and characterization, 1990–2021

**Figure 1.**
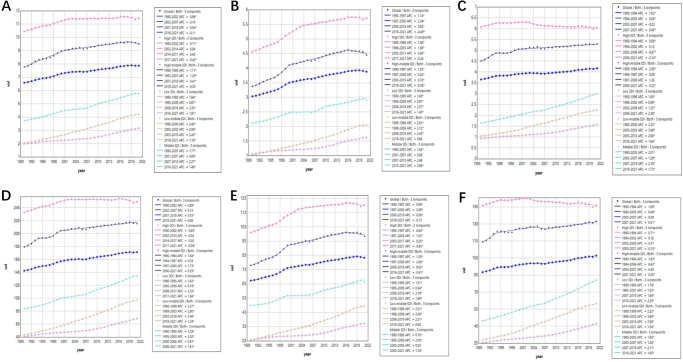
Trends in joint point regression analyses of ASRs (per 100 000 population) of all cancers attributable to high metabolic risk from 1990 to 2021 for each SDI region. (A) Metabolic risk-attributable mortality for all cancers; (B) high-volume plasma glucose-attributable mortality for all cancers; (C) high BMI mortality for all cancers; (D) all cancers attributable to metabolic risk DALYs; (E) all cancers attributable to high-volume plasma glucose risk DALYs; and (F) all cancers attributable to high BMI risk DALYs. ASRs, age-standardized rates; SDI, socio-demographic index; DALYs, disability-adjusted life years.

Joint observational regression analyses revealed differences in metabolism-related tumor burden across various SDI regions worldwide. As illustrated in Figure 1, all cancer attributions (high BMI, high FPG, and metabolic risk) were higher in high SDI regions compared to other SDI regions, while all cancer attributions were lower in low SDI regions than in other SDI regions. For mortality rates linked to high FPG and high BMI, the medium SDI region, the medium-low SDI region, and the low SDI region exhibit year-to-year increases. After 2019, only the mortality rates attributable to high FPG demonstrate a significant decreasing trend in the high SDI region and the medium-high SDI region.

**Figure 2.**
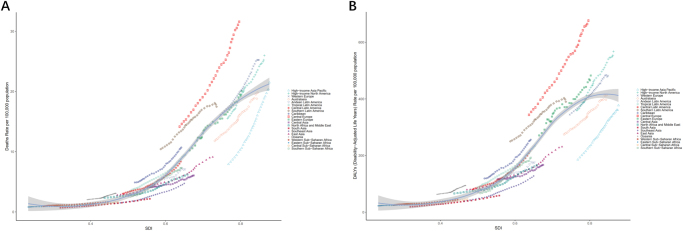
Trends in metabolic risk attributable to all cancers in 21 SDI regions, 1990–2021. (A) Trends in mortality rates (per 100 000 population) attributable to metabolic risk for all cancers in the 21 SDI regions, 1990–2021; (B) trends in DALYs (per 100 000 population) attributable to metabolic risk for all cancers in the 21 SDI regions, 1990–2021. SDI, socio-demographic index; DALYs, disability-adjusted life years.

Between 1990 and 2021, the low and medium SDI regions had the highest growth rates among deaths and DALYs, with AAPC values of 2.488 per 100 000 (95% UI: 2.359−2.616; *P* < 0.001) and 2.480 per 100 000 (95% UI: 2.382−2.579; *P* < 0.001) (Table 1). In the meantime, the distribution of growth rates for both of these conditions was the same for males, females, and all genders, 53%, 49%, and 51%, respectively (Table 2). Analysis of the 21 SDI regions showed that mortality rates and DALYs tended to increase annually and were significantly higher than expected in all regions of the globe, with the highest rates in Central Europe, followed by high-income North America and Western Europe (Fig. 2).

**Table 1:** **Trend analysis of the global burden of metabolism-related cancer** **in different income regions, 1990–2021**

	Global			High SDI			High-middle SDI			Low SDI			Low-middle SDI			Middle SDI		
	Female	Male	Both	Female	Male	Both	Female	Male	Both	Female	Male	Both	Female	Male	Both	Female	Male	Both
Deaths																		
AAPC	0.810	0.575	0.433	0.714	0.273	0.021	0.938	0.670	0.527	1.380	1.524	1.729	**2.281**	**2.346**	**2.488**	1.122	1.479	1.936
Lower CI	0.720	0.511	0.358	0.650	0.172	0.123	0.753	0.526	0.300	1.303	1.461	1.670	**2.187**	**2.247**	**2.359**	0.832	1.308	1.870
Upper CI	0.901	0.640	0.507	0.777	0.375	0.082	1.123	0.813	0.754	1.457	1.587	1.789	**2.376**	**2.444**	**2.616**	1.413	1.651	2.002
*P*-value	<0.001	<0.001	<0.001	<0.001	<0.001	0.688	<0.001	<0.001	<0.001	<0.001	<0.001	<0.001	**<0.001**	**<0.001**	**<0.001**	<0.001	<0.001	<0.001
DALYs																		
AAPC	0.718	0.563	0.503	0.563	0.192	0.011	0.809	0.618	0.477	1.303	1.465	1.650	**2.273**	**2.350**	**2.480**	1.048	1.468	1.916
Lower CI	0.627	0.493	0.423	0.499	0.090	−0.137	0.641	0.394	0.302	1.242	1.414	1.577	**2.187**	**2.272**	**2.382**	0.859	1.372	1.856
Upper CI	0.808	0.633	0.583	0.627	0.294	0.158	0.977	0.842	0.652	1.363	1.516	1.723	**2.359**	**2.428**	**2.579**	1.238	1.564	1.977
*P*-value	<0.001	<0.001	<0.001	<0.001	<0.001	0.889	<0.001	<0.001	<0.001	<0.001	<0.001	<0.001	**<0.001**	**<0.001**	**<0.001**	<0.001	<0.001	<0.001

AAPC, annual average percentage change; CI, confidence interval; DALYs, disability-adjusted life years; SDI, socio-demographic index.

The data of SDI area in Low-middle was bolded.

**Table 2:** Metabolic risk burden of all cancers associated with different income regions of the world from 1990 to 2021

Year	Deaths (1/100 000)			DALYs (1/100 000)		
	Male	Female	Both	Male	Female	Both
Global						
1990	6.26	6.86	6.59	141.43	158.83	150.59
	(1.77, 11.00)	(2.02, 11.86)	(1.91, 11.45)	(42.39, 244.70)	(48.16, 270.87)	(45.52, 257.42)
2005	7.43	7.46	7.43	165.91	171.86	168.80
	(2.06, 13.00)	(2.01, 13.01)	(2.03, 12.96)	(49.39, 285.95)	(47.59, 294.79)	(48.50, 289.40)
2021	7.99	7.80	7.87	177.99	181.20	179.36
	(2.21, 14.08)	(2.05, 13.53)	(2.12, 13.76)	(53.19, 310.91)	(49.52, 311.01)	(51.23, 312.41)
Cumulative change (%)	0.22	0.12	0.16	0.21	0.12	0.16
High SDI						
1990	10.89	10.15	10.45	248.08	235.24	240.81
	(3.16, 18.94)	(2.74, 17.73)	(2.93, 18.28)	(75.53, 426.86)	(63.15, 407.64)	(68.98, 415.15)
2005	12.33	10.71	11.41	277.38	247.13	260.78
	(3.41, 21.34)	(2.67, 18.79)	(3.01, 19.90)	(81.38, 474.84)	(61.91, 427.71)	(71.11, 448.64)
2021	12.29	10.75	11.44	271.22	245.44	257.44
	(3.38, 21.26)	(2.67, 18.78)	(3.00, 19.79)	(79.79, 461.28)	(63.39, 421.84)	(71.24, 439.98)
Cumulative change (%)	0.11	0.06	0.09	0.09	0.04	0.06
High-middle SDI						
1990	7.72	7.90	7.77	181.10	194.18	187.54
	(2.12, 13.59)	(2.52, 13.48)	(2.34, 13.33)	(52.62, 314.44)	(64.48, 327.01)	(59.06, 318.49)
2005	9.71	9.02	9.25	221.09	216.19	217.39
	(2.67, 17.10)	(2.61, 15.39)	(2.62, 15.99)	(64.66, 386.64)	(64.68, 365.35)	(64.51, 372.95)
2021	10.09	9.10	9.51	232.81	216.05	223.53
	(2.77, 18.13)	(2.61, 15.48)	(2.67, 16.38)	(68.41, 413.27)	(63.71, 365.20)	(66.28, 383.11)
Cumulative change (%)	0.23	0.13	0.18	0.22	0.10	0.16
Middle SDI						
1990	3.49	4.00	3.75	84.08	99.70	91.84
	(0.89, 6.26)	(1.16, 7.02)	(1.04, 6.65)	(24.33, 147.78)	(31.02, 170.95)	(28.07, 160.25)
2005	4.49	4.80	4.46	106.97	118.37	112.60
	(1.22, 8.01)	(1.27, 8.36)	(1.25, 8.18)	(31.70, 186.90)	(33.20, 205.08)	(32.97, 195.71)
2021	5.75	5.85	5.78	138.36	146.58	142.40
	(1.61, 10.39)	(1.55, 10.36)	(1.56, 10.30)	(42.53, 245.82)	(40.93, 256.20)	(41.68, 248.38)
Cumulative change (%)	0.39	0.32	0.35	0.39	0.32	0.36
Low-middle SDI						
1990	1.67	2.47	2.07	40.66	62.63	51.44
	(0.47, 2.89)	(0.77, 4.28)	(0.62, 3.55)	(12.62, 68.66)	(20.49, 107.65)	(16.71, 86.74)
2005	2.46	3.48	2.98	59.90	88.15	74.09
	(0.70, 4.36)	(0.98, 6.05)	(0.86, 5.24)	(18.40, 103.46)	(26.04, 150.90)	(22.58, 128.14)
2021	3.57	4.82	4.22	86.73	122.70	105.22
	(1.05, 6.37)	(1.22, 8.50)	(1.14, 7.51)	(27.20, 152.23)	(32.20, 213.80)	(30.15, 184.79)
Cumulative change (%)	**0.53**	**0.49**	**0.51**	**0.53**	**0.49**	**0.51**
Low SDI						
1990	1.63	2.36	1.99	38.38	60.21	48.98
	(0.47, 2.92)	(0.66, 4.20)	(0.57, 3.53)	(12.16, 66.70)	(17.96, 106.01)	(15.28, 84.52)
2005	1.89	2.79	2.34	44.39	70.00	57.12
	(0.54, 3.36)	(0.72, 4.89)	(0.64, 4.11)	(13.71, 76.39)	(19.66, 121.07)	(16.96, 98.29)
2021	2.52	3.79	3.16	58.64	94.21	76.63
	(0.71, 4.45)	(0.85, 6.88)	(0.80, 5.76)	(17.44, 102.80)	(22.67, 168.50)	(20.98, 137.54)
Cumulative change (%)	0.35	0.38	0.37	0.35	0.36	0.36

DALYs, disability-adjusted life years; SDI, socio-demographic index

The data of SDI area in Low-middle was bolded.

### Future projections of the global cancer burden from metabolic diseases

**Figure 3.**
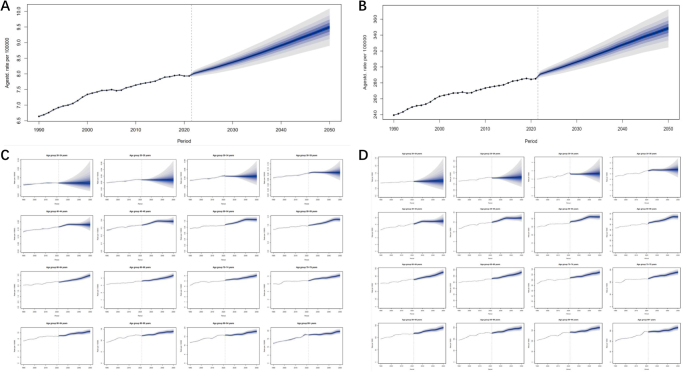
Future global age-standardized and age-attributable metabolic risk mortality and DALY rates for all cancers, projected to 2050. (A) Global age-standardized mortality rates for all cancers attributable to metabolic risk, projected to 2050 (per 100 000 population); (B) global mortality from metabolic risk attributable to all cancers at 20 years of age, projected to 2050 (per 100 000 population); (C) global standardized age-attributable metabolic risk DALYs for all cancers, projected to 2050 (per 100 000 population); (D) global DALYs for attributable metabolic risk for all cancers at 20 years of age, projected to 2050 (per 100 000 population). DALYs, disability-adjusted life years.

The burden of cancer due to metabolic diseases is increasing every year and may have important implications for human health in the future. In this context, we further projected future cancer incidence rates. Projections applying the BAPC model showed that global age-standardized and age-attributable metabolic risk mortality projections for all cancers showed a yearly increasing trend from 2021 to 2050 (Fig. 3). By 2030, mortality rates and DALYs are projected to rise to 13.25 (95% UI: 12.80–13.71) and 306.31 (95% UI: 294.92–317.70) cases per 100 000 population, respectively. By 2050, this prevalence is projected to reach 15.03 cases per 100 000 (95% UI: 13.91–16.14) and 348.50 cases per 100 000 (95% UI: 320.24–376.75) (Table 3). In addition, analysis of cancer incidence rates in different age groups showed a high incidence of cancer among those aged 40–59 years, with a gradual decrease in risk with increasing age. Mortality rates and DALYs are projected to increase with age in the 60+ age group, with an increasing trend from year to year. By 2050, mortality rates and DALYs for the 95+ age group will be at their highest. The older age group of 95+ will exhibit the highest mortality rates and DALYs, estimated to be approximately 196.22 per 100 000 and 1660.75 per 100 000, respectively (Table 3, Fig. 3).

**Table 3:** Future global standardized ages for all cancers in 2030 and 2050, as well as ASRs (per 100 000 population) 95% UI projections of attributable metabolic risk mortality and disability-adjusted life year rates for individuals aged 40 and above in 2050

	Death	DALYs
	ASRs	ASRs
Time		
2030	13.25 (12.80–13.71)	306.31 (294.92–317.70)
2050	15.03 (13.91–16.14)	348.50 (320.24–376.75)
Age group in 2050 (years)		
40–44	1.82 (1.35–2.30)	90.42 (70.94–109.90)
45–49	3.86 (3.31–4.40)	171.63 (151.35–191.91)
50–54	10.55 (9.65–11.45)	422.04 (385.91–458.17)
55–59	18.34 (16.89–19.80)	643.97 (589.43–698.51)
60–64	29.68 (27.36–31.99)	902.63 (826.40–978.86)
65–69	43.61 (40.24–46.98)	1124.37 (1029.55–1219.19)
70–74	60.68 (56.01–65.35)	1294.06 (1185.04–1403.08)
75–79	81.71 (75.44–87.98)	1405.06 (1286.77–1523.35)
80–84	104.38 (96.38–112.38)	1413.41 (1294.41–1532.41)
85–89	140.27 (129.53–151.01)	1501.04 (1374.65–1627.43)
90–94	179.43 (165.70–193.17)	1639.26 (1501.21–1777.32)
95+	196.22 (181.18–211.26)	1660.75 (1520.82–1800.69)

ASRs, age-standardized rates; DALYs, disability-adjusted life years; UI, uncertainty interval.

## Discussion

Our study provides a comprehensive overview of the increasing global burden of cancers linked to high metabolic risk factors. In addition to the previously documented regional and socioeconomic disparities, our analysis uniquely emphasizes key gaps that have not been addressed in the literature, particularly concerning the elderly population^[4,6]^. Older adults are at significantly greater risk due to age-related physiological changes such as impaired glucose tolerance, chronic inflammation, and decreased metabolic efficiency, rendering them particularly vulnerable to cancers resulting from metabolic dysregulation. Regional variations in dietary patterns, levels of physical activity, and healthcare infrastructure largely influence the differences in cancer burden^[7]^.

Additionally, the main reasons for growth in low and medium SDI regions are changes in dietary habits, widespread consumption of high-sugar processed foods, and the lack of primary screening for metabolic diseases such as uncontrolled diabetes. Our study also corroborates previous studies. Metabolic disorders were also differentially associated with specific cancer types. High BMI is associated with colorectal cancer, and high FPG is associated with liver cancer^[8,9]^.

We should recognize the value of healthcare interventions that combine modern medical technologies with traditional diagnostic and therapeutic modalities to increase population awareness of routine healthcare and metabolic testing and to promote early detection and intervention of cancer through improved preventive care and professional counseling^[10]^. For areas with high SDI, it is recommended to focus on the health management of the elderly. Specifically, in future clinical practice, it is recommended that metabolic indicators be screened annually in the community for people aged 60 and above. For areas with low and moderate SDI, it is recommended to focus on primary prevention and accessibility. It is recommended to promote the distribution of portable blood glucose meters in primary care settings to foster a general understanding of the population’s metabolic profile. Future research should continue to elucidate the biological mechanisms that lead to the development of cancer, investigate region-specific risk factors, and evaluate the effectiveness of targeted intervention strategies for demographic and socioeconomic backgrounds^[11]^. Integrating and utilizing emerging biomedical strategies to manage the burden of cancer due to metabolic dysfunction. In addition, our study provides valuable predictions of future cancer incidence, emphasizing the importance of preventive population health measures in the future cancer burden^[12]^. Future studies should address these limitations by incorporating more detailed and diverse data, exploring potential mediating influences, and examining clinically effective interventions.

## Conclusion

This study highlights the growing global cancer burden from cancers associated with high metabolic risk. There is an urgent need for healthcare interventions focused on screening and systematic prevention and treatment for populations at high metabolic risk. We recommend that low- and middle-income countries prioritize the establishment of metabolic cancer screening systems to address and manage the growing global cancer health problem effectively.

## Data Availability

None.
